# Unanticipated remission of primary hyperparathyroidism following cinacalcet

**DOI:** 10.1210/jcemcr/luag014

**Published:** 2026-03-27

**Authors:** Sara Ramadan, Shirin Haddady

**Affiliations:** Department of Medicine, Boston Medical Center Brighton, Boston University Chobanian & Avedisian School of Medicine, Boston, MA 02135, USA; Department of Medicine, Division of Endocrinology, Diabetes and Weight Management, Boston Medical Center, Boston University Chobanian & Avedisian School of Medicine, Boston, MA 02118, USA

**Keywords:** primary hyperparathyroidism, cinacalcet, hypercalcemia, parathyroid adenoma, medical management, calcium-sensing receptor

## Abstract

Primary hyperparathyroidism (PHPT) is the most common etiology of hypercalcemia in an ambulatory setting and often arises from adenomatous growth of one (occasionally more than one) parathyroid gland. Patients with severe and symptomatic hypercalcemia, renal insufficiency, nephrolithiasis, osteoporosis, and younger than 50 are candidates for surgical removal of parathyroid adenoma(s). Medical treatment with a calcimimetic agent is offered to improve severe hypercalcemia when surgery is refused or considered unsafe. Here, we report a 68-year-old man with severe hypercalcemia due to PHPT and coronary artery disease who had to undergo percutaneous coronary intervention and stent placement. Surgery for PHPT was delayed for 6 months, and he received cinacalcet to lower serum calcium. He developed hypocalcemia 6 months later. After discontinuation of cinacalcet, his serum calcium remained normal. Ultrasonography of the anterior neck showed again a hypoechoic lesion posterior to the right thyroid lobe (presumed to be a parathyroid adenoma) but with smaller dimensions. This case highlights a rare but clinically significant phenomenon: the possibility of durable remission and anatomical regression of parathyroid adenoma following cinacalcet administration.

## Introduction

Primary hyperparathyroidism (PHPT) is one of the most common endocrine disorders, ranking just behind diabetes mellitus and thyroid disorders in worldwide prevalence [1]. Up to 1% of adults develop PHPT, with incidence increasing in older women [2]. Substantial racial differences in the prevalence of PHPT are recognized. African Americans, particularly women, have the highest risk for PHPT and tend to present with more severe biochemical disease. The reasons for these disparities are multifactorial, including genetic, metabolic, and healthcare access factors [3]. The etiology of the vast majority of PHPT cases (80-85%) is attributed to a solitary parathyroid adenoma, while hyperplasia, multiple adenomas, and parathyroid carcinoma represent less frequent etiologies [4, 5]. The disorder is characterized by autonomous secretion of parathyroid hormone (PTH), driving persistent hypercalcemia and increased risk for nephrolithiasis, osteoporosis, neuropsychiatric symptoms, and cardiovascular disease [1, 3, 6].

Surgical removal of the parathyroid adenoma(s) offers cure rates exceeding 95% with well-documented improvements in bone mineral density, renal function, and nephrolithiasis, and overall quality of life [1, 5, 7]. Surgical intervention is recommended for patients younger than 50 and those with nephrolithiasis, renal insufficiency, osteoporosis, and serum calcium levels that are 1 unit above the normal range or higher [8]. However, many patients, particularly elderly or those with significant comorbidities, could be poor surgical candidates or decline operative intervention for personal reasons [4, 8]. In such cases, medical management would be beneficial to improve hypercalcemia and to reduce fracture risk due to osteoporosis. Such treatments were traditionally regarded as a temporary approach. However, a few recent case reports described the possibility of a durable normocalcemia even after cessation of medical therapy with calcimimetic agent, cinacalcet [8, 9].

Cinacalcet acts as a positive allosteric modulator of the calcium-sensing receptor (CaSR) on parathyroid cells, suppressing PTH secretion and lowering serum calcium [9-11]. It is approved by the US Food and Drug Administration for the treatment of secondary hyperparathyroidism from end-stage renal disease, parathyroid carcinoma, and severe hypercalcemia in patients with PHPT in whom surgery is not feasible [10, 12].

The present case describes a patient with PHPT and severe hypercalcemia who experienced not only biochemical remission but also a marked reduction in parathyroid adenoma volume following a brief course of treatment with cinacalcet.

## Case presentation

A 68-year-old South Asian male was referred to endocrinology clinic in our center for evaluation of hypercalcemia following an abnormal routine laboratory screening by his primary care provider shortly after immigration to the United States. His medical history included coronary artery disease (CAD) and coronary artery bypass grafting (CABG) 12 years prior, hypertension, and dyslipidemia. He denied a personal history of nephrolithiasis, peptic ulcer disease, and thyroid disorders, as well as a family history of PHPT, hypercalcemia, and nephrolithiasis. He was not a smoker. His medications included aspirin 81 mg daily, metoprolol 50 mg twice daily, atorvastatin 20 mg daily, and lisinopril 10 mg daily. He reported chronic fatigue persisting for several years and mild generalized musculoskeletal pain, previously attributed to aging and exertional shortness of breath. He acknowledged mild constipation and occasional mood lability but denied polyuria, polydipsia, confusion, or memory loss. Physical examination revealed an elderly man with a body mass index of 31.7 kg/m^2^. His exam was unremarkable except for bilateral pitting edema in the lower extremities.

## Diagnostic assessment

**Figure 1:**
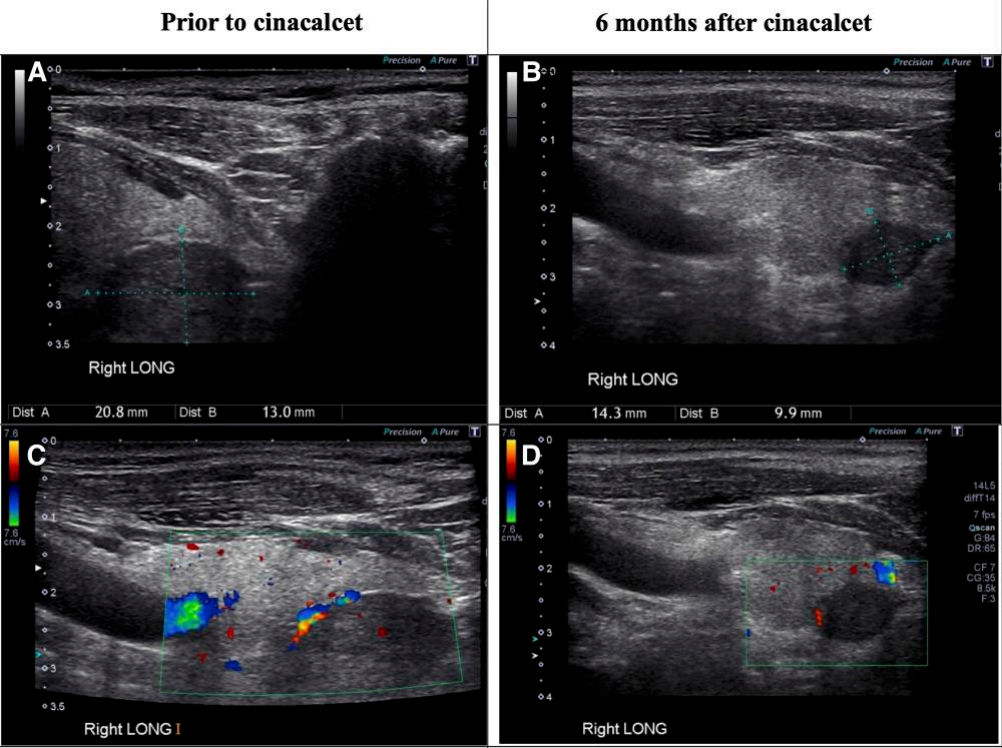
Ultrasound image of presumed parathyroid adenoma prior to initiating cinacalcet and after 6 months of treatment with cinacalcet. (A) 2.3 × 1.7 × 3.0 cm mass posterior to right lobe prior to cinacalcet. (B) 1.4 × 1.0 × 1.1 cm mass posterior to right lobe 6 months after cinacalcet. (C) Hypoechoic with clear borders and feeding vessel. (D) Doppler images of the same mass.

Laboratory evaluation (Table 1) showed serum calcium concentration of 12.0 mg/dL (Système International [SI]: 2.99 mmol/L; reference range: 8.5-10.5 mg/dL and 2.1-2.5 mmol/L), intact PTH level of 451 pg/mL (SI: 47.9 pmol/L; reference range: 15-65 pg/mL and 1.6-6.9 pmol/L), serum phosphate of 2.2 mg/dL (SI: 0.71 mmol/L; reference range: 2.3-4.7 mg/dL and SI: 0.74-1.52 mmol/L), 25-hydroxy vitamin D level of 34 ng/mL (SI: 85 nmol/L; reference range: 30-100 ng/mL and 75-250 nmol/L), serum creatinine of 1.02 mg/dL (SI: 90 µmol/L; reference range: 0.6-1.3 mg/dL and SI: 53-115 µmol/L), estimated glomerular filtration rate of >60 mL/min/1.73 m^2^, and alkaline phosphatase level of 153 IU/L (SI: 153 U/L; reference range: 120 IU/L). Serum albumin and magnesium were within normal limits. Dual-energy X-ray absorptiometry revealed osteopenia with a lumbar spine *T*-score of 0.33 and a femoral neck *T*-score of −2.0. Because his serum calcium level was more than 1 unit above the normal range, he was considered a candidate for surgical intervention. In an attempt to localize a parathyroid adenoma(s), a high-resolution ultrasonography in endocrinology clinic was performed, which showed no thyroid gland abnormalities. However, a well-demarcated, homogeneous, hypoechoic 2.3 × 1.7 × 3.0 cm mass with peripheral vascular flow resembling a feeding blood vessel was identified posterior to the inferior right thyroid lobe (Figure 1).

**Table 1:** Biochemical values prior to and 6 months after cinacalcet treatment and 1 month after stopping the medication

Parameter	Prior to cinacalcet	6 Months on cinacalcet	1 Month after stopping cinacalcet	Reference range
Calcium	12.1 mg/dL (2.99 mmol/L)	6.8 mg/dL (1.7 mmol/L)	9.1 mg/dL (2.3 mmol/L)	8-10.5 mg/dL (2.1-2.6 mmol/L)
Creatinine	1.02 mg/dL (90 µmol/L)	1.12 mg/dL (99 µmol/L)	1.2 mg/dL (106 µmol/L)	0.7-1.3 mg/dL (62-115 µmol/L)
PTH	451 pg/mL (47.9 pmol/L)	14 pg/mL (1.5 pmol/L)	44 pg/mL (4.6 pmol/L)	11-90 pg/mL (1.1-9.0 pmol/L)
25 (OH) D	34 ng/mL (85 nmol/L)	31 ng/mL (78 nmol/L)	39 ng/mL (98 nmol/L)	30-100 ng/mL (75-250 nmol/L)
Phosphorus	2.2 mg/dL (0.71 mmol/L)	4.4 mg/dL (1.42 mmol/L)	3.3 mg/dL (1.07 mmol/L)	2.8-4.1 mg/dL (0.9-1.3 mmol/L)

Abbreviations: 25 (OH) D, 25-hydroxyvitamin D; PTH, parathyroid hormone.

## Treatment

Due to a prior history of CAD and CABG, bilateral peripheral edema, and complaints about exertional dyspnea, he was referred to cardiology clinic prior to surgery. He underwent a positron emission tomography scan, which showed a large-sized, severe, reversible perfusion defect of the entire anterior wall, basal anteroseptum, and the anterolateral wall consistent with ischemia. Coronary angiography demonstrated severe multivessel disease, including critical stenoses of the left main, left anterior descending, left circumflex, and right coronary arteries, with subtotal or total occlusions in multiple branches. He underwent percutaneous coronary intervention and stent placement, and parathyroid surgery was postponed for 6 months until completion of dual antiplatelet therapy. Due to severe and symptomatic hypercalcemia, medical treatment with cinacalcet 30 mg daily was initiated, which was later titrated to 30 mg twice daily. Cholecalciferol 2000 IU daily was continued to maintain vitamin D sufficiency.

## Outcome and follow-up

After 6 months of starting cinacalcet, he returned to endocrinology clinic for a regular follow-up visit and complained of a new onset paresthesia for several days. Laboratory evaluation revealed serum calcium concentration of 6.8 mg/dL (SI: 1.70 mmol/L), intact PTH level was 14 pg/mL (SI: 1.5 pmol/L), and serum creatinine concentration was 1.12 mg/dL (SI: 99 µmol/L) (Table 1). Cinacalcet was discontinued, and oral calcium carbonate 1000 mg 3 times a day was started. The patient's symptoms resolved gradually, and 1 month later, repeat laboratory studies showed complete resolution of hypocalcemia and low PTH (Table 1). Remarkably, a repeat neck ultrasound showed a mass inferior to the right thyroid lobe with similar characteristics as the previous study, but with a size of 1.4 × 1.0 × 1.1 cm, representing >50% reduction of the original size (Figure 1). Twelve months later, his serum calcium concentration was 9.8 mg/dL (SI: 2.45 mmol/L), and intact PTH level was 51 pg/mL (SI: 5.4 pmol/L) while maintained on vitamin D supplementation (cholecalciferol 2000 IU daily). Almost 3 years later, total serum calcium concentration was 8.9 mg/dL (SI: 2.22 mmol/L).

## Discussion

As a CaSR agonist, cinacalcet lowers PTH levels and corrects hypercalcemia. Evidence from meta-analyses demonstrates that ∼90% of PHPT patients achieve normal serum calcium, regardless of age, sex, or disease severity [13]. Response rates are highest in those with marked hypercalcemia (serum calcium ≥12 mg/dL), where symptom burden is greatest and immediate intervention is needed [14]. Despite its proven efficacy in normalizing serum calcium levels, cinacalcet typically does not achieve complete PTH normalization in most patients with PHPT. This difference reflects the fundamental distinction between cinacalcet's biochemical mechanism, which relies on CaSR-mediated suppression of PTH synthesis and secretion from existing parathyroid tissue, and surgical parathyroidectomy, which physically removes the pathological adenomatous tissue that serves as the primary source of excess PTH production [14]. The effect of cinacalcet on serum calcium continues as long as the medication is taken, and often hypercalcemia returns if the treatment is paused.

However, recent literature has reported a small number of cases in which cinacalcet induced not only biochemical remission but also anatomical regression or disappearance of parathyroid adenoma [15, 16]. The latter effect was thought to be due to parathyroid apoplexy, acute necrosis or hemorrhagic infarction of an adenoma during cinacalcet therapy [2, 16, 17]. The prescribed dose of cinacalcet is different among the published case reports. Table 2 shows a comparison of reported cases with adenoma regression after cinacalcet.

**Table 2:** **Cases of adenoma regression after cinacalcet [** 17-21 **]**

Study/case	Patient demographics	Baseline characteristics	Cinacalcet duration/dose	Outcome after treatment	Mechanism proposed	Adenoma size	Long-term follow-up
Current case report	68-year-old male	Calcium: 12.1 mg/dL (2.99 mmol/L) PTH: 451 pg/mL (47.9 pmol/L)	6 months therapy, 30 mg BID	Claimed remission after discontinuation	Adenoma regression	23 mm initially → 14 mm after remission	Normal Ca/PTH at 1 year follow-up
Di Dalmazi et al (2018) [17]	80-year-old male	Calcium: 11.9 mg/dL (2.98 mmol/L) PTH: 681 pg/mL (72.5 pmol/L)	17 months, up to 180 mg daily	Severe hypocalcemia, adenoma necrosis/apoplexy	Cinacalcet-induced parathyroid apoplexy/infarction	9 mm initially → 17 mm during apoplexy	Normal Ca/PTH at 1 year follow-up
Nguyen et al (2020) [18]	59-year-old male with prior failed parathyroidectomy	Calcium: 10.9 mg/dL (2.72 mmol/L) PTH: 205.6 pg/mL (21.9 pmol/L)	Short-term exposure, low dose	Transient hypocalcemia and then sustained eucalcemia	Apoptosis of remaining adenoma cells	No clear radiographic adenoma identified	>3 years eucalcemic off supplements
Krcma (2024) [19]	64-year-old female with severe pancreatitis	Calcium: 13.6 mg/dL (3.4 mmol/L) PTH: 740 pg/mL (78.7 pmol/L)	Up to 180 mg 3 times daily	9-year sustained remission	High-dose calcimimetic induced adenoma regression	10 mm initially → 5 mm after remission	9 years sustained remission
Wahid et al (2023) [20]	58-year-old male with CKD	Calcium: 12.9 mg/dL (3.23 mmol/L) PTH: 238 pg/mL (25.2 pmol/L)	Brief therapy, 30 mg BID	Hypocalcemia, adenoma necrosis, vocal cord palsy	Cinacalcet-induced adenoma necrosis	38 mm initially → 4 mm with partial necrosis	Sustained normocalcemia postsurgery
Minezaki et al (2021) [21]	Nine patients (1M:8F), mean age 58.1 ± 7.2 years	Calcium: mean 11.38 mg/dL (2.84 mmol/L) PTH: mean 126.5 pg/mL (13.5 pmol/L)	6 months, 50 mg daily	29% adenoma size reduction	Direct adenoma size reduction via CaSR modulation	Mean 10-25 mm initially → no significant change reported	6 months only

Abbreviations: BID, twice daily; Ca, calcium; CaSR, calcium-sensing receptor; CKD, chronic kidney disease; PTH, parathyroid hormone.

Several mechanisms have been proposed to explain these outcomes. Cinacalcet's activation of the CaSR not only suppresses PTH synthesis and secretion but also, particularly in secondary hyperparathyroidism, suppresses parathyroid cell proliferation and increases apoptosis [10, 11, 15]. Upregulation of CaSR expression and restoration of normal calcium signaling in adenomatous tissue may contribute to the shrinkage or involution of the mass [21]. In vitro and in vivo studies support the ability of cinacalcet to reduce cell proliferation and induce apoptosis [11, 15, 21]. Case series analyzing adenoma size before and after cinacalcet treatment have noted a reduction in adenoma volume of up to 29% after 6 months of therapy [13, 20].

This case report portrays similar findings, demonstrating both clinical and radiographic remission 6 months after initiating cinacalcet. The patient's sustained normocalcemia, normal PTH, and volume reduction of the parathyroid adenoma on imaging argue for a durable disease-modifying effect. The case contributes to the growing literature suggesting that cinacalcet may occasionally induce durable PHPT remission, possibly through mechanisms involving parathyroid adenoma regression, necrosis, or apoptosis. However, such outcomes remain rare and unpredictable, requiring close monitoring for hypocalcemia and long-term follow-up to evaluate response durability.

The sustained normocalcemia and regression of the adenoma in this patient raise the question of whether remission resulted from cinacalcet therapy or spontaneous infarction, a rare but recognized cause of “auto-parathyroidectomy” [22]. Spontaneous remission of PHPT due to adenoma infarction or hemorrhage has been reported since 1946, typically attributed to rapid tumor growth exceeding vascular supply, causing ischemia and necrosis. Kovacs and Gay [23] described 12 cases with variable presentations, ranging from neck pain, palpable mass, or acute hypocalcemia to entirely asymptomatic resolution. Lucas et al [24] noted that recurrence can occur months to years later, with the longest reported interval being 7 years. Larger adenomas (≥2 cm) are more susceptible to infarction. Cinacalcet may also contribute to adenoma involution via complementary mechanisms. Experimental studies demonstrate dose- and time-dependent apoptosis of human parathyroid cells and reversal of hyperplasia through CaSR activation in animal models. Clinically, cinacalcet reduces gland volume in secondary hyperparathyroidism; whether these effects occur in PHPT, or trigger infarction in poorly perfused large adenomas, is speculative but biologically plausible [18].

Distinguishing cinacalcet-induced remission from spontaneous infarction is challenging. Spontaneous infarction often presents acutely with neck pain, dysphagia, or hypocalcemia, whereas cinacalcet-induced apoptosis typically evolves gradually over weeks to months. Our patient had no neck pain, and imaging showed a >50% adenoma reduction (from 2.3 × 1.7 × 3.0 to 1.4 × 1.0 × 1.1 cm) with preserved echotexture.

There are limitations to case reports of this nature. The rarity of prolonged remission, the absence of histologic confirmation (in the absence of surgery or biopsy), and potential for confounding factors (such as spontaneous adenoma infarction or unrecognized autoimmune phenomena) must all be considered. Controlled prospective trials of cinacalcet for curative intent in PHPT are lacking. The long-term impact of calcimimetic therapy on bone health and nephrolithiasis, especially in the context of persistently elevated PTH, has not been fully elucidated [13, 14, 18]. Generalizability to wider populations and durability of remission beyond several years remain unknown.

The markedly elevated PTH of 451 pg/mL (∼7× upper limit of normal), in the context of preserved renal function and adequate vitamin D, raised concern for parathyroid carcinoma despite a serum calcium of 12.0 mg/dL, which is elevated but below levels typically seen in malignancy [25]. Parathyroid carcinoma is rare (<1% of PHPT) and difficult to distinguish preoperatively, with clinical clues including calcium >14 mg/dL, PTH >3× normal, symptomatic hypercalcemia, palpable neck mass, and imaging showing large, irregular, or heterogeneous lesions. The ultrasound characteristics of the parathyroid adenoma in this case were homogeneous, well-demarcated, with a peripheral feeding vessel. In addition, the patient remained eucalcemic, which would be unlikely if the original etiology of his hypercalcemia was parathyroid carcinoma.

Recent guidelines from major endocrine societies and expert groups emphasize surgery as the preferred, definitive management for PHPT, particularly in those with symptoms, end-organ involvement, or at higher risk for complications [5, 7, 12]. Cinacalcet may be appropriately reserved for patients who are not surgical candidates or decline surgery and have severe hypercalcemia, but with close monitoring for complications and evaluation for disease progression or remission on therapy [10, 12].

## Learning points

- Rarely in patients with PHPT, cinacalcet may induce biochemical and anatomical remission—potentially reversing hypercalcemia and restoring a normal level of calcium even after stopping the medication.
- Vigilant monitoring for hypocalcemia is essential while cinacalcet is prescribed for severe hypercalcemia in a patient with PHPT.
- Further research is required to understand the true frequency, dose relationship, and pathogenesis of durable remission of hypercalcemia with cinacalcet.

## Contributors

All authors made individual contributions to the authorship. S.H. involved in the diagnosis and clinical management of the patient. S.R. contributed to the conception of the work, acquisition and interpretation of clinical and laboratory findings, and preparation of ultrasound images. All authors reviewed and approved the final draft.

## Data Availability

Some or all datasets generated during and/or analyzed during the current study are not publicly available but are available from the corresponding author on reasonable request.
